# Statistical Analysis of the Effectiveness of Seawalls and Coastal Forests in Mitigating Tsunami Impacts in Iwate and Miyagi Prefectures

**DOI:** 10.1371/journal.pone.0158375

**Published:** 2016-08-10

**Authors:** Roshanak Nateghi, Jeremy D. Bricker, Seth D. Guikema, Akane Bessho

**Affiliations:** 1 School of Industrial Engineering and Division of Environmental and Ecological Engineering, Purdue University, West Lafayette, IN, United States of America; 2 International Research Institute of Disaster Science, Tohoku University, Sendai, Japan; 3 Department of Industrial and Operations Engineering, University of Michigan, Ann Arbor, MI, United States of America; 4 Undergraduate program in Architectural Design, Maryland Institute College of Art, Baltimore, MD, United States of America; Osaka University Graduate School of Medicine, JAPAN

## Abstract

The Pacific coast of the Tohoku region of Japan experiences repeated tsunamis, with the most recent events having occurred in 1896, 1933, 1960, and 2011. These events have caused large loss of life and damage throughout the coastal region. There is uncertainty about the degree to which seawalls reduce deaths and building damage during tsunamis in Japan. On the one hand they provide physical protection against tsunamis as long as they are not overtopped and do not fail. On the other hand, the presence of a seawall may induce a false sense of security, encouraging additional development behind the seawall and reducing evacuation rates during an event. We analyze municipality-level and sub-municipality-level data on the impacts of the 1896, 1933, 1960, and 2011 tsunamis, finding that seawalls larger than 5 m in height generally have served a protective role in these past events, reducing both death rates and the damage rates of residential buildings. However, seawalls smaller than 5 m in height appear to have encouraged development in vulnerable areas and exacerbated damage. We also find that the extent of flooding is a critical factor in estimating both death rates and building damage rates, suggesting that additional measures, such as multiple lines of defense and elevating topography, may have significant benefits in reducing the impacts of tsunamis. Moreover, the area of coastal forests was found to be inversely related to death and destruction rates, indicating that forests either mitigated the impacts of these tsunamis, or displaced development that would otherwise have been damaged.

## Introduction

**Fig 1:**
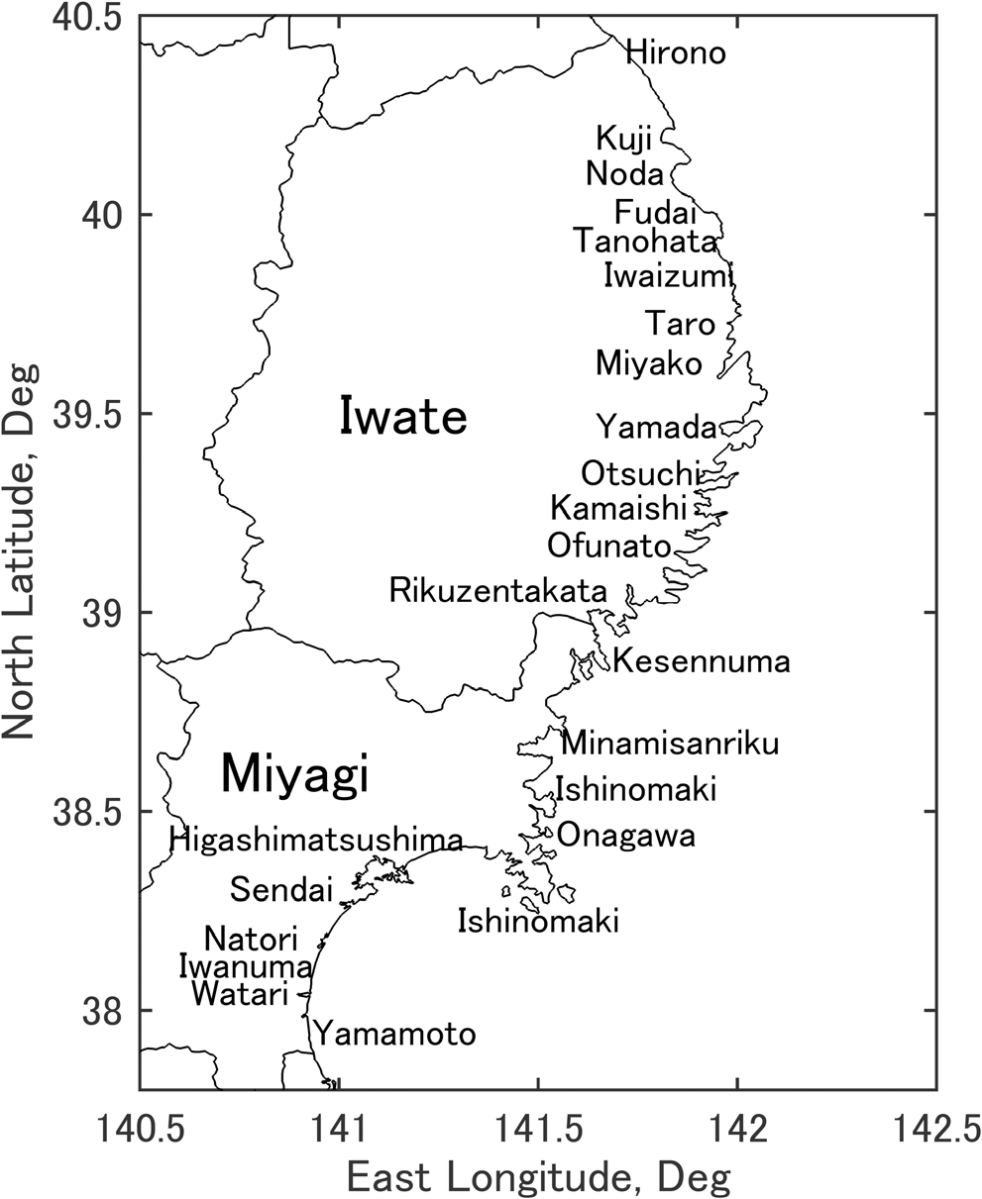
Map of coastal municipalities in Iwate and Miyagi Prefectures in 2011. In addition, the municipalities of Shiogama, Tagajo, Rifu, Shichigahama, and Matsushima lie between Sendai and Higashimatsushima. Figure created by the authors using prefecture boundary data from [18] and MATLAB software.

Over the past century, the Pacific coast of Japan’s Tohoku region (Fig 1) has experienced repeated offshore tsunamis. In 1896, a tsunami occurred after the magnitude 8.5 Meiji Sanriku Earthquake at 7:32 pm Japan Standard Time (JST) on June 15, 1896. The 1933 tsunami was generated by the magnitude 8.4 Showa Sanriku Earthquake on March 3, 1933 at 2:30 am JST. The magnitude 9.5 Great Chilean Earthquake (the only far-field event considered in this analysis) sent waves across the Pacific Ocean, arriving along the coast of Tohoku approximately 22 hours later, on May 24, 1960 at about 2:30 am JST. The magnitude 9.0 Great East Japan Earthquake occurred on March 11, 2011 at 2:46 pm JST, generating a tsunami which arrived along the Sanriku (Iwate and northern Miyagi) coast as quickly as 20 minutes later and along the Sendai Plain (southern Miyagi) coast about 1 hour later [1]. The source and impact of each of these events are summarized in [2,3].

After the 1933 event, a limited number of cities in Iwate Prefecture constructed “hard” tsunami defense structures along the coast specifically to protect lives and property from tsunamis. After the 1960 event, the number of projects for this purpose increased rapidly [4]. In order to protect lives, “soft” countermeasures have also been implemented; an example of this is the nationwide tsunami warning system, which began operation in 1952 [5]. Coastal forests have also been used for tsunami mitigation in the region since the 17^th^ century, though their effectiveness during large events is an active topic of research [6].

The Japanese government’s reconstruction plan [7,8] relies heavily on seawalls (encompassing flood walls, embankments, and river-mouth gates) and bay-mouth breakwaters. From FY2011-2015, reconstruction projects were given a total budget of 25 trillion yen (about $200 billion) by the central government [9]. From FY2016-2021, the planned reconstruction budget is 6.5 trillion yen (about $55 billion), with 22 billion yen to be contributed by Fukushima, Miyagi, and Iwate Prefectures [10].

However, detractors conjecture that hard coastal structures cause a sense of complacency in residents [11,12], leading to lower evacuation rates and to the tendency to develop residences in hazardous low-lying areas such as the town of Taro. Some residents oppose seawall construction because of possible negative effects on ocean views and tourism [13]. Aldrich and Sawada [14], who analyzed municipal level data from the 2011 tsunami, contend that seawalls had no effect on preventing casualties, and that the $1.6 billion Kamaishi breakwater (the world’s tallest) “crumbled upon impact” [12] and failed to provide the town with any protection. Rather, they cite social factors such as community cohesiveness as the main factor affecting mortality in 2011.

Contrarily, Tomita et al. [15] showed that the Kamaishi and Ofunato breakwaters, even in their post-tsunami damaged state, significantly reduced the extent of each town flooded by the tsunami, mitigated flow speed and depth in the inundated areas, and delayed tsunami arrival time long enough to aid the evacuation of residents. Furthermore, others cite the example of Fudai, where a large river-mouth gate saved the town from destruction [16]. To address this debate in a quantitative fashion, we implement a statistical analysis of the effectiveness of hard coastal defense structures on damage to dwellings and loss of lives using municipal and sub-municipal level historical data from the 1896, 1933, 1960, and 2011 tsunamis. The role of coastal forests in mitigating [6] vs. exacerbating damage [17] is examined in like manner.

## Data Collection and Visualization

S1 Table presents historic data on casualties and damage in each coastal municipality of Miyagi and Iwate Prefectures due to the 1896, 1933, 1960, and 2011 tsunamis, as well as sub-municipal data for 2011. Table 1 lists sources for the data shown in S1 Table. Due to mergers of municipalities throughout the 20^th^ century, the number of municipalities has decreased since the 1896 and 1933 events. Elevations are reported with respect to the Tokyo Peil (TP) vertical datum.

**Table 1:** Data Sources.

	1896 Miyagi	1896 Iwate	1933 Miyagi	1933 Iwate	1960 Miyagi	1960 Iwate	2011 Miyagi	2011 Iwate	2011 sub-municipal
**Tsunami runup elevation**	[45] [79] [78]	[45] [79] [78]	[45] [79] [78]	[45] [79] [78]	[56] [78]	[56] [78]	[57] [58] [59] [60] [78]	[51] [57] [58] [59] [60] [78]	[78]
**Seawall elevation**	N/A	N/A	N/A	N/A	N/A	[48]	[66]	[71] [72]	[70]
**Municipal area**	[81]	[81]	[81]	[81]	[75]	[75]	[43]	[43]	[46] [80] [55] [67] [68] [76] [74] [61] [65] [73] [69]
**Inundated area**	[45]	[45] [50]	[45]	[45]	[63]	[50]	[42]	[42] [54] [65]	
**Population before tsunami**	[62]	[47]	[75]	[75]	[75]	[75]	[75]	[75]	
**Number of people killed**	[62]	[47]	[45]	[45]	[56] [63]	[49] [56]	[64]	[51]	
**Number of dwellings before tsunami**	[62]	[47]	[45]	[45]	[63]	[52]	[41]	[41] [52]	
**Number of dwellings destroyed**	[62]	[47]	[45]	[45]	[56] [63]	[49] [56]	[53] [57] [58] [59] [60] [64]	[53] [44] [57] [58] [59] [60]	
**Forest area**	[77]	[77]	[77]	[77]	[77]	[77]	Google Earth	Google Earth	Google Earth

In this study, the number “dead” includes both those reported as “dead” and those reported as “missing”. Dwellings “destroyed” includes the sum of the number of dwellings reported as “swept away”, “collapsed”, and “completely damaged”. “Death rate” or “mortality” is defined as the ratio of the number dead in each municipality (or sub-municipality) divided by the total population of the municipality (or sub-municipality). Likewise, “damage rate” is the number of dwellings destroyed in the municipality divided by the total number of dwellings in the municipality. Other studies (i.e., [14]) define mortality differently, with the denominator containing only the population of the municipality within the area inundated by the tsunami. However, records from historical tsunami events (1896, 1933, and 1960) do not contain information on population and number of dwellings within the inundated area, so total municipal area is used for all events in order to maintain consistency.

In S1 Table, seawall elevations in 2011 are listed as crest elevation before the 2011 event, though many locations experienced various modes of failure, such as scour (especially during drawdown), parapet toppling, and overtopping [19,20,21]. The bay mouth breakwaters listed in S1 Table were also damaged. Despite the damage these coastal facilities experienced, [15,22] showed these structures delayed tsunami arrival time by several minutes, thus affording residents more time to evacuate. The structures also reduced overland inundation extent, depth, and flow speed, reducing the number of homes destroyed. Since damaged structures provided protection, and since time and extent of damage are not precisely known, the statistical analysis below is carried out using the pre-tsunami values of seawall crest height and bay mouth breakwater presence. In many municipalities, maximum and minimum values of seawall heights are listed; this represents non-uniformity of seawall heights in those municipalities. Tsunami elevations listed in S1 Table represent the maximum run-up in each municipality as recorded in each source of Table 1.

In addition to census, municipal, and damage data, topography is listed in S1 Table as a variable affecting vulnerability to tsunami. Topography is parameterized as either coastal “plain” or “ria”. For the most part, the coast south of Ishinomaki (Fig 1) is a broad, open plain, while the ria coast north of Ishinomaki consists of very narrow valleys surrounded by high mountains. [23] showed the importance of local topography (distance to high ground) and warning time on mortality in 2011. [24] also cited topography as a major factor determining mortality in 2011, and found that mortality varied widely even among cities with similar damage rates. Topography also controls the manner in which tsunamis impact coastal towns; ria towns see tsunamis arrive as a gradually to rapidly rising water level [25], while tsunamis can impact the coastal plain as bores (vertical walls of water). [26] showed the effect of topography on runup, the primary parameter used to indicate local tsunami height in this analysis.

**Fig 2:**
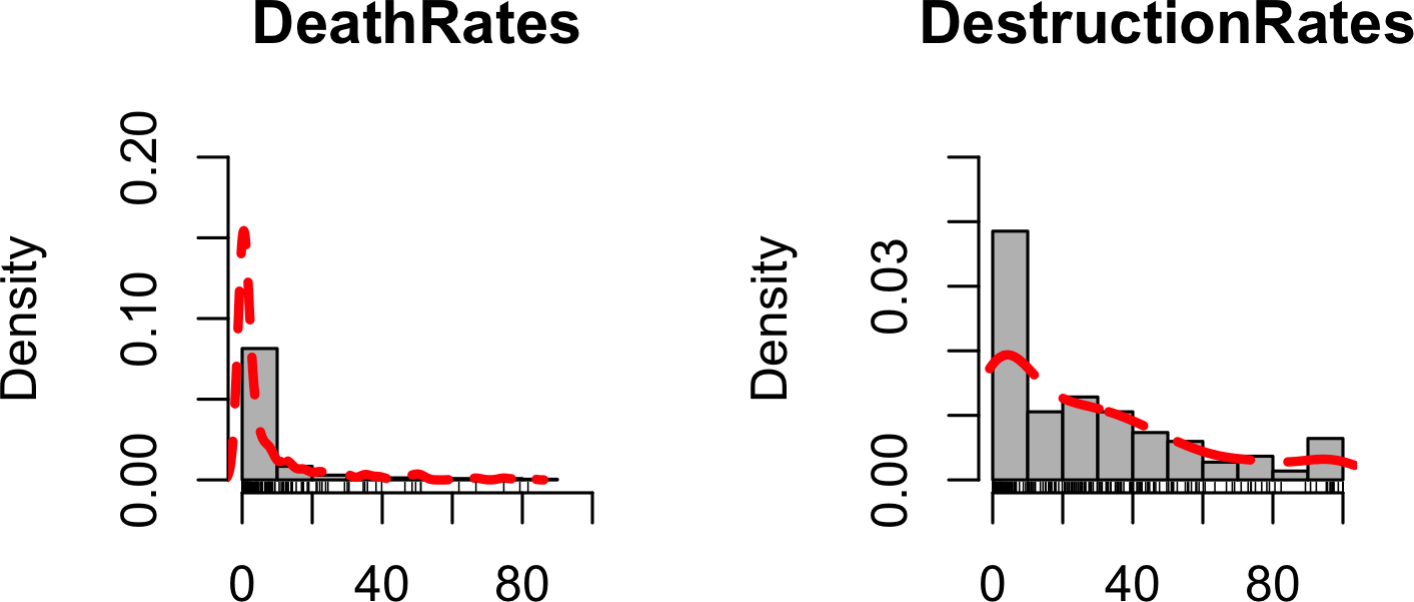
Histograms of total death and destruction rates caused by tsunamis of 1896, 1933, 1960 and 2011. The red dashed lines represent kernel density plots of death and destruction rates respectively.

Histograms of total death and destruction rates are shown in Fig 2 below. It can be seen that both distributions are positively skewed with mortality rates being more heavily skewed than destruction rates.

**Fig 3:**
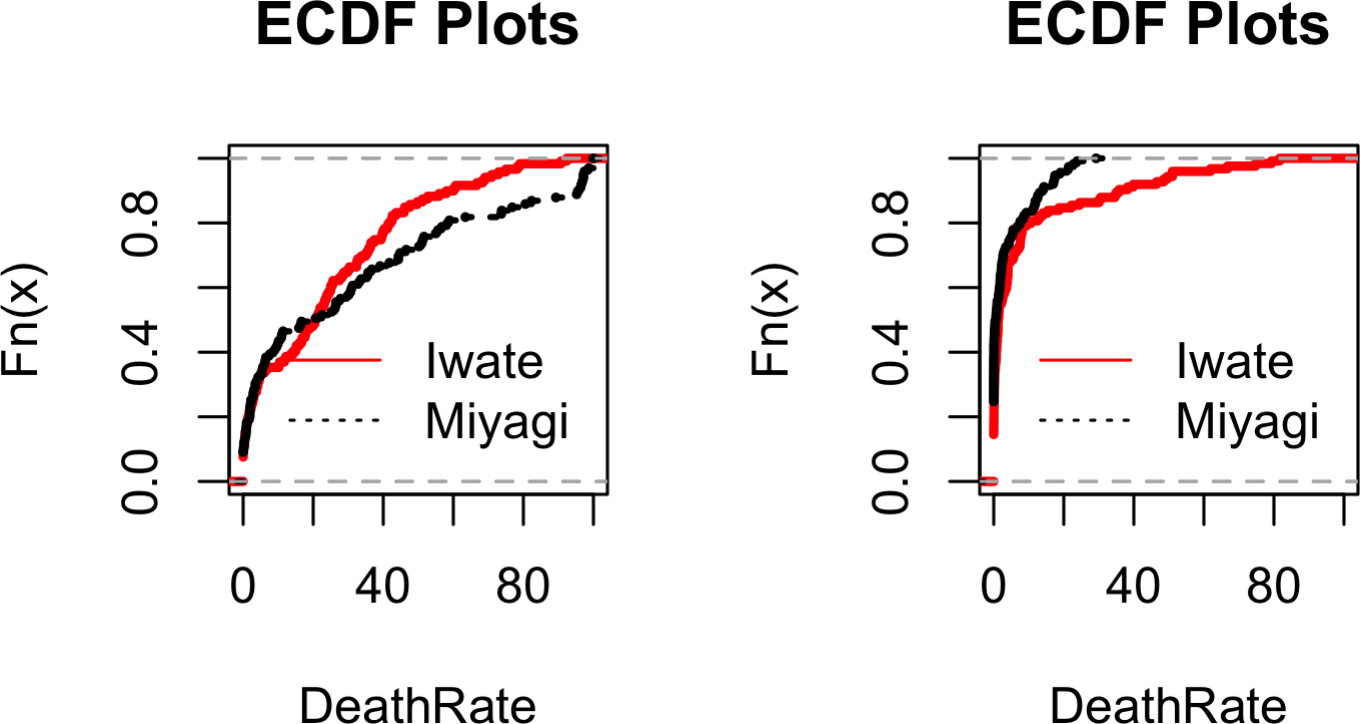
Empirical cumulative distribution function (ecdf) plots of total damage and destruction rates for prefectures of Iwate and Miyagi.

The empirical cumulative distribution function (ecdf) plots of total damage and destruction rates for each prefecture have been plotted in Fig 3. Table 2 summarizes the ranges for the death and damage rates for the two prefectures.

**Table 2:** Ranges of percentage destruction and death rates in Miyagi and Iwate Prefectures.

Variable	Prefecture	Min.	1^st^ Q.	Median	Mean	3^rd^ Q.	Max.
Destruction Rate (%)	Miyagi	0.00	2.57	20.74	31.72	51.45	100.00
Destruction Rate (%)	Iwate	0.00	2.82	20.63	24.24	38.40	92.39
Death Rate (%)	Miyagi	0.00	0.00	0.67	3.96	4.80	29.23
Death Rate (%)	Iwate	0.00	0.13	1.29	9.50	7.63	81.62

It can be seen both from Fig 3 and Table 2 that while destruction rates were only slightly higher in Iwate than in Miyagi, maximum death rates have been substantially higher in Iwate compared to Miyagi; this is mostly a result of heavy casualties in Iwate during the 1896 tsunami (S1 Table).

**Fig 4:**
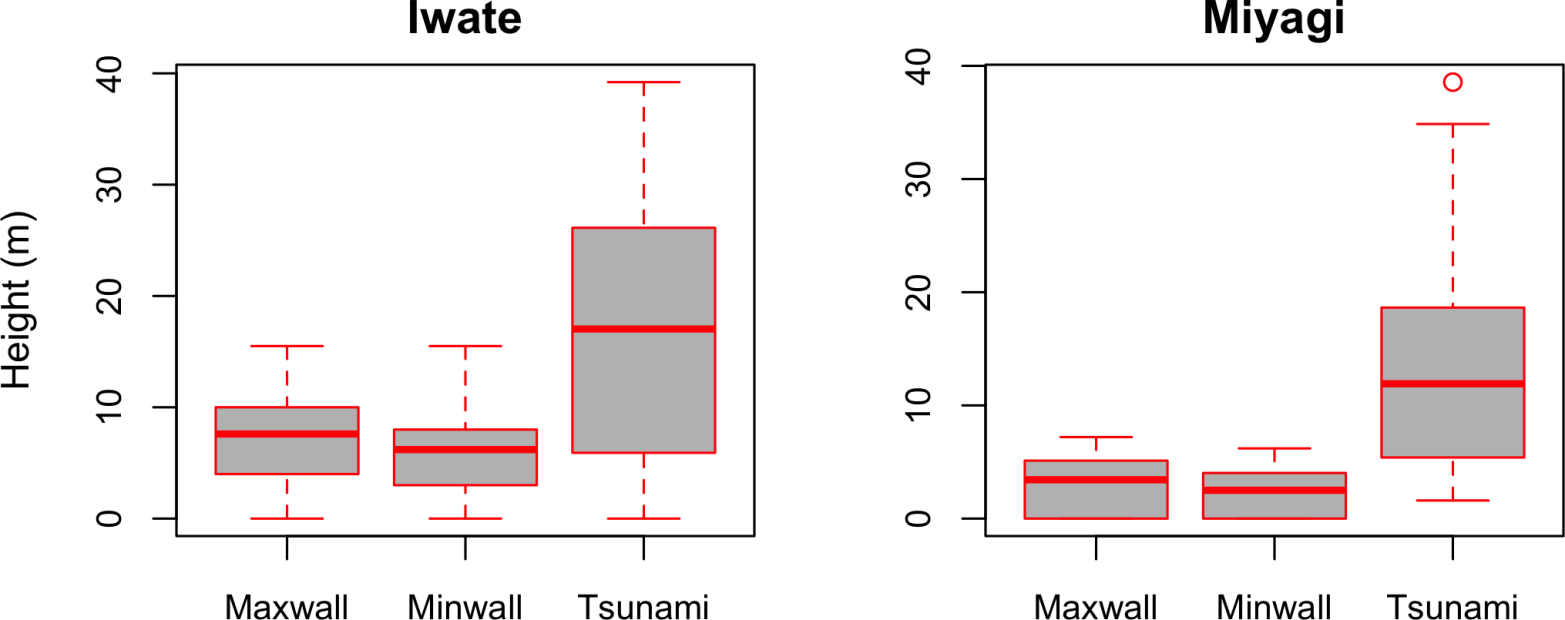
Seawall heights and tsunami heights in Iwate and Miyagi Prefectures.

Furthermore, it is interesting to compare the seawall characteristics and tsunami heights for each prefecture. As can be seen in Fig 4, seawalls as well as tsunami heights are on average higher in Iwate than Miyagi.

**Fig 5:**
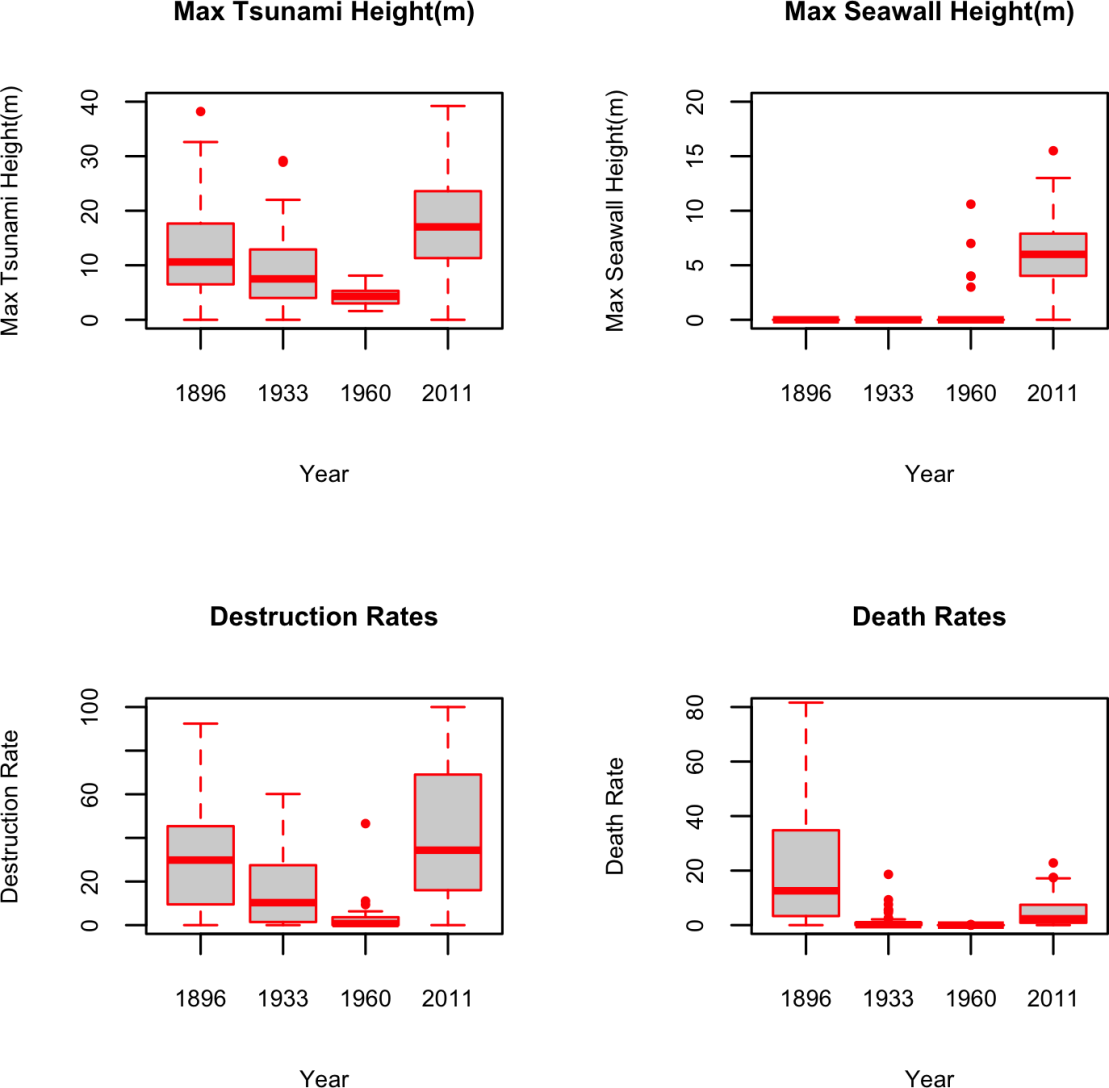
Boxplots of maximum tsunami height, max seawall height, death and damage rates for the years of 1896, 1933, 1960, 2011.

Fig 5 depicts the summary statistics of maximum tsunami and seawall heights, as well as death and damage rates during 1896, 1933, 1960 and 2011. It can be seen that the most extreme maximum tsunami heights were observed in 2011, and the far-field 1960 event was the mildest in terms of its tsunami height. Also, except for only 6 cases in 1960 (5 of which were in Iwate Prefecture), no seawalls existed during the 1960 and earlier events. It is also interesting to note that while the destruction rates were not significantly different between 1896 and 2011, the death rates were much higher in 1896 compared to 2011. This is in large part due to the fact that the 1896 earthquake did not produce violent ground motion (similar to the more recent events described in [27] and [28]), so many residents did not evacuate [2].

**Fig 6:**
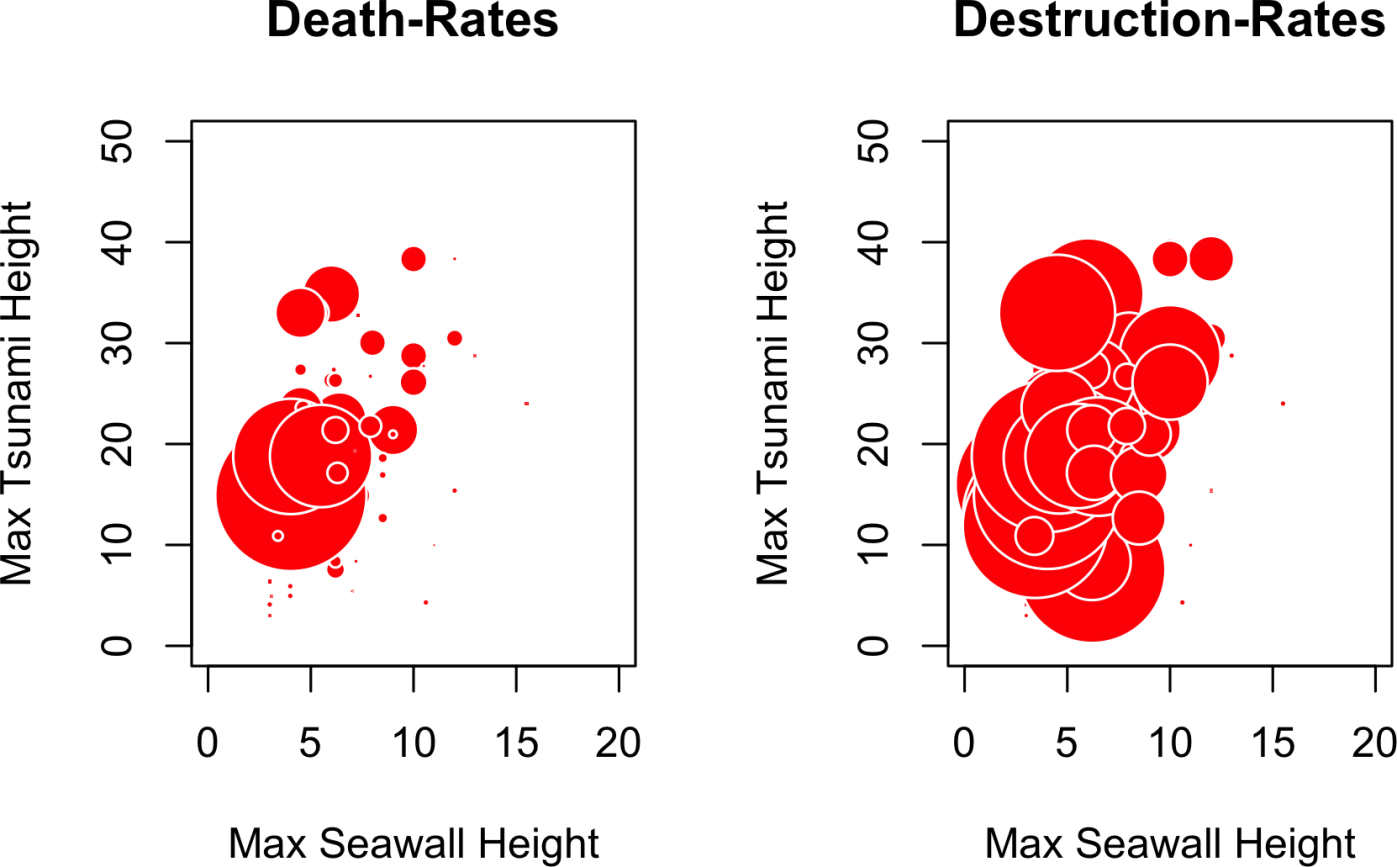
Plots of maximum tsunami heights versus maximum seawall heights for death and destruction rates.

The bubble charts in Fig 6 depict the impact of maximum tsunami heights and maximum seawall heights on damage and destruction rates, for cases in which seawalls existed. The size of the bubbles corresponds to percentage death and destruction rates respectively. One would expect to see the largest bubbles clustered in the upper left-hand side of the plot. While this is mostly the case, particularly for death rates, it is apparent from the plots that tsunami heights and seawalls heights alone do not depict a full picture.

**Fig 7:**
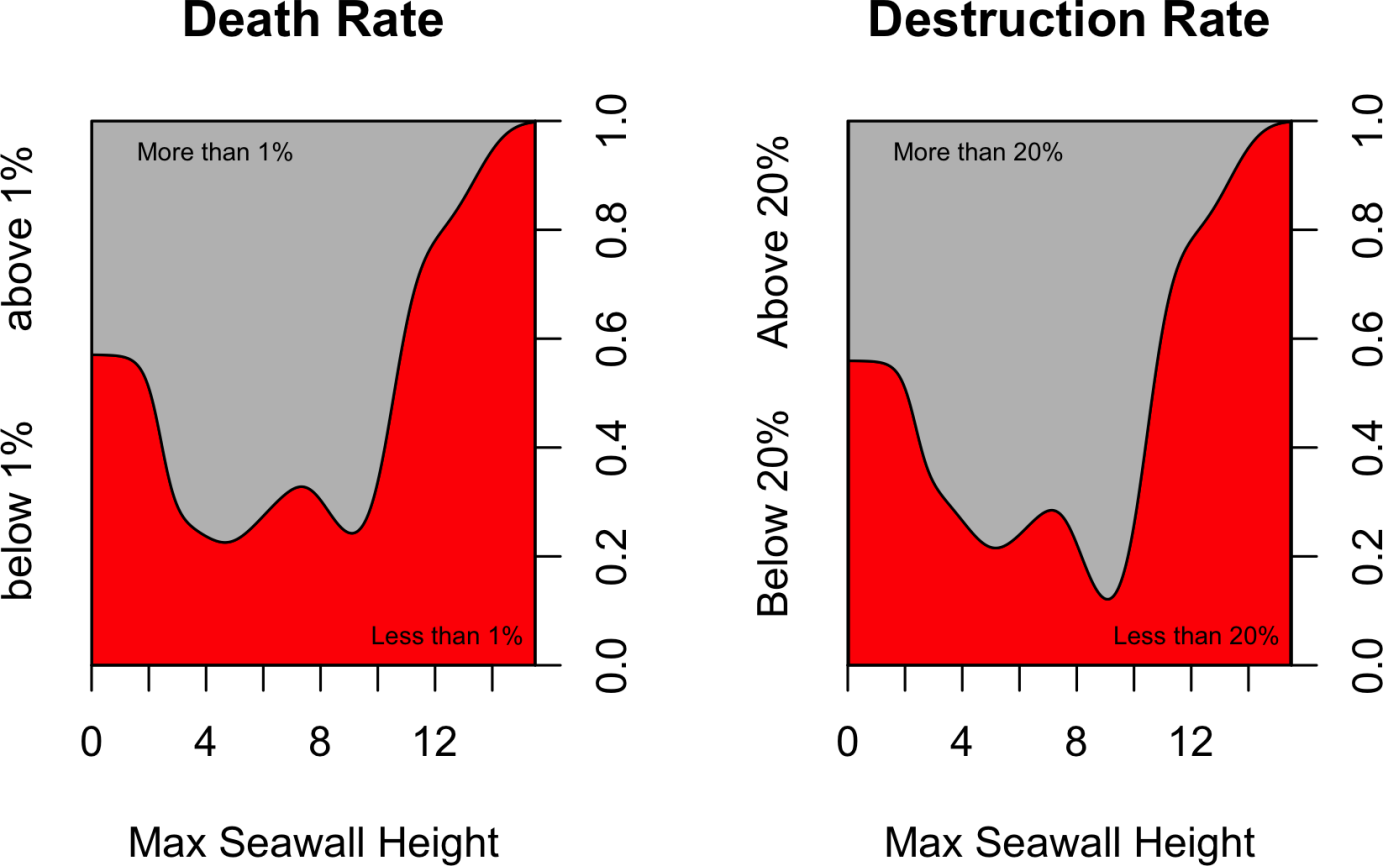
Conditional density plots for death and damage rates versus maximum seawall heights. Here the response variables are binary, representing whether death and damage rates are above or below their median values. For a given seawall height, a greater proportion of red means lower death or damage rates, while a greater proportion of gray means higher death or damage rates.

Conditional density plots describe how the conditional distribution of a given categorical response variable changes as the explanatory variable changes. In Fig 7, the response variables represent whether death or damage rates are above or below their median values. The median death rates and destruction rates are around 1% and 20% respectively. Small seawalls (around 5 m high) are associated with a higher likelihood of death and destruction rates being above their median values (more destruction), while large seawalls are associated with higher likelihood of the death and destruction rates being below their median values (less destruction).

## Methods

A brief review of statistical learning methods relevant to analyzing the tsunami data is presented in this section. We begin by stating the distinction between supervised and unsupervised learning methods and discuss different classes of supervised learning methods, namely, parametric, semi-parametric and non-parametric methods. We then deliberate the details of the statistical models used in this paper. We conclude by discussing bias-variance trade-off and also the trade-offs between predictive accuracy and model interpretability.

### Supervised Learning Methods

Broadly speaking, statistical learning methods refer to a large pool of algorithms and tools used for data analysis. Statistical learning methods can be categorized into two groups of supervised (the focus of this paper) and unsupervised learning methods. Unlike unsupervised learning methods (ULM), in supervised learning methods (SLM) the observed target variable of interest (e.g. tsunami-induced damage rates in a given year) guides the learning process. Models can be developed to predict the target variable based on a range of input variables (e.g. tsunami height and sea-wall heights). The ultimate goal is developing a model that can best capture the relationship between the predictors and response and minimize the loss function (i.e. the difference between the observed and predicted values of the target variable).

The target variable of interest can be denoted as *Y* and the matrix of input variables as *X*. As mentioned earlier, supervised learning methods seek to approximate the unknown functional dependence of *X* and *Y* such that the specified loss function *L(y*,*f)* is minimized. This relationship can be summarized as the Eq 1 below:

$$\hat{f}(X)=Y\;and\;\hat{f}(X)=argmin\;L(y,f(x))$$ (1)

Linear and non-linear supervised statistical learning methods can be parametric, semi-parametric or non-parametric. In this paper, we develop a range of models to best predict tsunami-induced death and damage rates.

### Parametric models

In parametric methods, assumptions are made about the shape of function *f* that relates the input variables to response. The advantage is that by assuming a functional form, the problem of estimating an arbitrary p-dimensional function is reduced to estimating a set of parameters. The disadvantage of parametric models is that the assumed shape usually does not match the true unknown function *f*.

#### Generalized linear models (GLM)

The term generalized linear models was coined by Nelder and Wedderburn in early 1970s [29]. A GLM extends ordinary linear regression by allowing the response to take a probability distribution other than the normal distribution, and be related to the predictors through a link function. In a generalized linear model, the outcome variable *Y* is assumed to be generated from the family of exponential distribution (such as Gaussian, binomial, Poisson, gamma, or inverse-Gaussian) as shown in Eqs 2 and 3 below

$$Y_{i}∼f_{Y_{i}}(y_{i})$$ (2)

$$f_{Y_{i}}(y_{i})=exp\{\frac{y_{i}\theta_{i}-b(\theta_{i})}{a(\phi )}+c(y_{i},\phi )\}$$ (3)

where *θ* and *ϕ* are location and scale parameters. The parameter(s) of the response *(μ)* is then tied to the linear combination of the input variables through a link function *(g)*. This can be summarized in the equation below

$$E[Y_{i}]=\mu_{i}$$ (4)

$$g(\mu_{i})=x_{i}^{\prime }\beta$$ (5)

### Semi-parametric models

Semi-parametric models lie at the fuzzy boundary between parametric and non-parametric techniques. Semi-parametric models offer more flexibility compared to parametric models and better interpretability compared to non-parametrics.

#### Generalized additive models (GAM)

Generalized additive models are non-linear extensions to generalized linear models [30]. Similar to a GLM model, the mean of the response variable is linked to the covariates via a link function. However, the linear functional form of combining the covariates is relaxed as shown in the Eq 6.

$$g(\mu_{i})=\sum s_{j}(x_{j})+\varepsilon$$ (6)

where *s*_*j*_ represent the smoothers applied over the input variables.

#### Multivariate adaptive regression splines (MARS)

MARS is a semi-parametric model that allows for local non-linearities and interaction effects which makes it suitable for modeling high-dimensional datasets [31]. A MARS model consists of sum-of-splines that allow the response to vary non-linearly with the input variables.

$$f(x)=\beta_{0}+\sum \beta_{m}h_{m}(X)+\varepsilon$$ (7)

where *h*_*m*_ represents the linear splines, *β*_*0*_ represents the intercept and *β*_*m*_ represents the vector of the coefficients. *β*_*m*_ coefficients are estimated by minimizing the sum of square errors.

### Non-parametric models

Non-parametric models do not make assumptions about the shape of the function *f*. Instead they use the data in novel ways to approximate it. While it has the advantage of not assuming unrealistic functional form and potentially better approximating the true function, it is very data intensive. Also, improved prediction comes at the cost of reduced interpretability which will be discussed later in this section.

#### Bayesian additive regression trees (BART)

BART is a Bayesian, tree-based approach. A BART model consists of the summation of *m* binary decision trees, each constrained by a prior that restrict each tree’s contribution to the final model, making each decision tree a ‘weak learner’ [32]. A BART model can be summarized using the equation below. The error term of the model is assumed to be normally distributed with a mean of 0 and constant standard deviation.

$$Y=\sum_{j=1}^{m}g(x;\;T_{j},M_{j})+\varepsilon \;\varepsilon ∼N(0,\sigma 2)$$ (8)

In the equation above, *g* is a single tree model with attributes: terminal nodes *T* and associated terminal node parameter *M*. The function *g*(*x*; *T*_*j*_,*M*_*j*_) assigns the mean values (μ) to the vector of x covariates. Model fit and inference in BART are achieved via Markov Chain Monte Carlo (MCMC) algorithm.

#### Random forest (RF)

Random forest is a non-parametric, tree-based ensemble data-miner [33]. An RF model consist of a large number of bootstrapped regression trees. There are two layers of randomness involved in developing an RF model. First the training data fitted to the trees are randomly sampled with replacement. Second, the subset of predictors selected at each node is also randomly selected. These levels of randomness result in reduced correlation levels amongst the generation trees. The final estimate is the result of averaging the predictions of all regression trees which results in a low-bias low variance final estimate.

#### Support vector machines

Support vector machines (SVM) is a powerful tool for big data analytics. Contrary to many data-mining methods that use greedy algorithms, SVM is a constrained optimization problem and does not suffer from local optima and handles high-dimensional data very well. In SVM-regression the input space is first mapped onto an m-dimensional feature space. A linear model is the constructed in this feature space. In other words, SVM regression involves developing a linear regression in a high dimensional feature space [34,35].

#### Gradient boosting machines (GBM)

This flexible data-mining technique was developed by [36]. In boosted trees method a greedy algorithm is used to minimize the overall loss function. GBM fits a sequence of single trees, using each tree to fit data variance not accounted for by the earlier sequence of trees. In other words, trees are iteratively added to the residuals of the preceding trees such that the additive weighted expansions of the trees yield great fit to the data.

### The Trade-Off Between Prediction Accuracy and Model Interpretability

As mentioned earlier, semi-parametric and non-parametric techniques are usually not constrained with the (often non-realistic) assumptions of more restrictive parametric models. While this offers the advantage of better approximating the systematic relationship between *X* and *Y* and better predictive power, the interpretability of these models are more limited than parametric models. However, there are methods available for conducting variable inference even for non-parametric models and one of these methods is outlined below

#### Partial Dependence Plots (PDP)

Partial dependencies show the influence of a covariate of interest, on the response, given that the effect of the rest of the covariates on the response are averaged out as shown in the equation below [37].

$$f_{s}(X_{s})=\frac{1}{N}\sum_{i=1}^{N}f(X_{s},x_{ic})$$ (9)

In the equation above, *S*_*X*_ stands for the variable for which the partial dependence plot is being calculated, and *x*_*ic*_ represents the remaining predictors used in the final model other than *X*_*s*_. In plotting the partial dependence plots, the *x* variable is varied (at small increments) while other variables are held constant; and the delta response is averaged across all the records. Partial dependence plots show the average change in the response variable as a function of variable *x*, while all other variables are held constant.

### Bias-Variance Trade-off

The generalization performance of a statistical model hinges on model’s capability to yield accurate predictions on an independent test sample. Bias-variance trade-off is central to ensuring minimized generalization error [30]. Cross-validation is the most widely used technique in estimating average generalization error and assessing bias-variance trade-off [37]. In this paper, we use the method of k-fold cross validation to estimate predictive accuracy of our models. K-fold cross-validation involves randomly subdividing the dataset into k subsets of almost equal sizes. In each iteration, the k^th^ subset is held-out; and the model is trained to the remaining subsets and the predictive accuracy is assessed based on the models performance on the k^th^ subset. This procedure is repeated multiple times to ensure that all data has been used at least once. In this paper we implemented 30 iterations of 10% hold-out cross validation. The reported mean absolute error (MSE) and mean absolute deviations (MAE) are the average errors across the 30 iterations.

## Results

This section summarizes the results our predictive models of damage rate and death rate respectively and discuss the importance of various factors such as tsunami heights, seawall heights, and coastal forest areas.

### Damage rates

This section summarizes the predictive performance of a series of models trained to our dataset. In these models, the response variable is damage rates and the independent variables include: the year of the event, the city population before the event, municipal area, maximum tsunami height, coastal forest area, presence of a bay-mouth breakwater, maximum and minimum seawall height, flooded area, prefecture, and topography. In order to deal with the missing data in our input variables, we used the Multivariate Imputation by Chained Equations (MICE) algorithm.

#### Out-of-sample prediction errors

As mentioned in the Methods section, we trained the data with a range of parametric and nonparametric models including: generalized linear model (GLM), generalized additive model (GAM), Bayesian additive regression trees (BART), random forest (RF), multi-variate regression splines (MARS), support vector machines (SVM) and gradient boosted trees (GBM). Table 3 summarizes the mean absolute error (MAE) and mean squared errors (MSE) and their associated standard errors (SE) for each of the models except for GLM and GAM since their errors were an order of magnitude larger than the rest of the models. The errors in Table 3 are based on 30 iterations of 10% random holdout cross-validations, where each time 10% of the data is randomly held-out and models are trained with the remaining 90%. The reported errors are calculated based on testing each model’s performance on the holdout samples. The ‘Null’ model below refers to not having a statistical model and using the mean as a predictor. It can be used to examine how much a given statistical model has contributed to explaining the variance in the response. We can see that the errors associated with RF are substantially lower than the ‘mean-only’ model indicating the effectiveness of the model in capturing the variance of damage rates.

**Table 3:** Out-of-sample predictive accuracy across different models.

Model	MSE	SE	MAE	SE
Null	813.1	54.9	23.6	0.7
BART	387.9	24.3	15.4	0.5
**RF**	**323.6**	**18.9**	**13.9**	**0.4**
MARS1	526.3	51.2	16.8	0.6
MARS2	512.6	55.1	16.5	0.6
MARS3	649.8	136.9	17.0	0.7
MARS5	690.1	202.7	16.6	0.7
SVM	582.2	43.2	17.8	0.7
GBM	755.9	51.4	22.7	0.7

The method of Random Forest (RF) outperformed all other models in terms of out-of-sample predictive accuracy. The difference between the MSE and MAE values of RF and all other models were statistically significant (based on the Wilcoxon signed-rank test).

**Fig 8:**
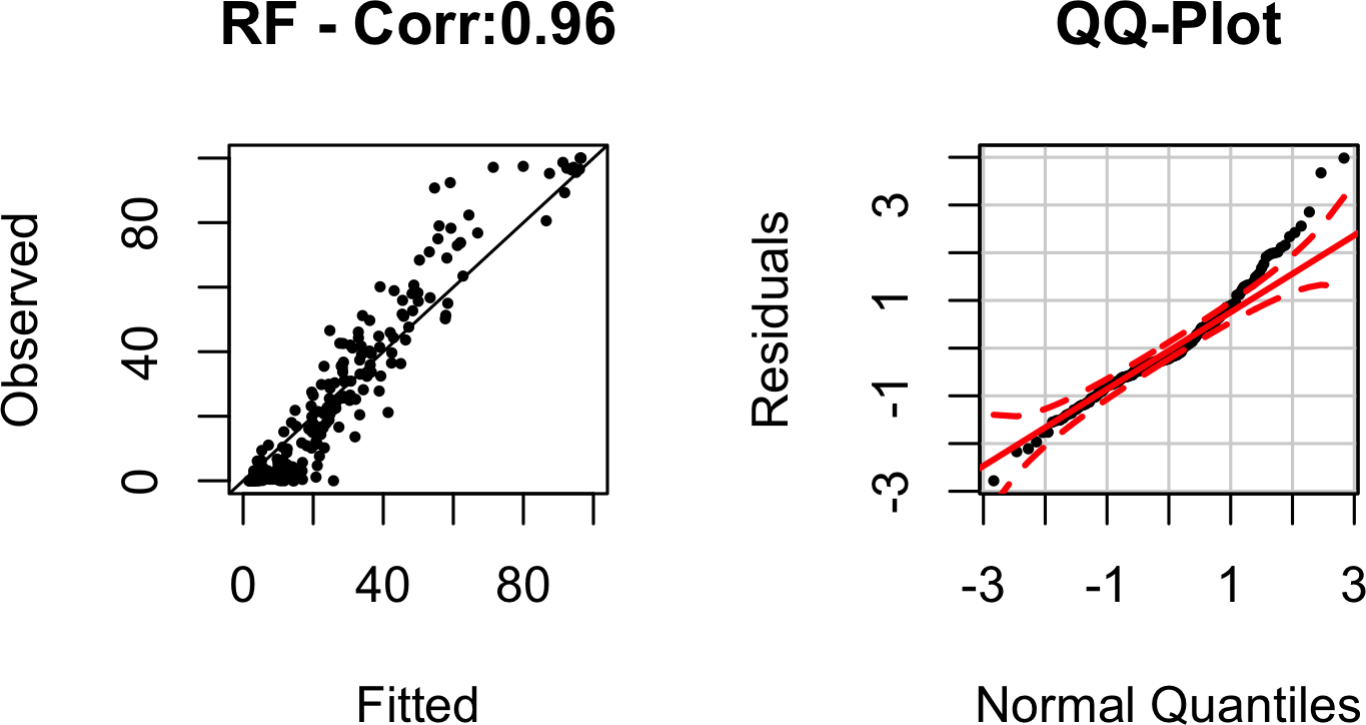
Plot of observed versus fitted values of destruction rate for methods of RF (correlation 0.96) together with the normal Q-Q plot. The red dashed lines in the QQ-plot represent 95% confidence intervals.

To examine how well our selected final model (RF) fitted the data, we plotted observed destruction rates versus our model’s estimates along with the model’s residuals as shown in Fig 8. The correlation between the observed values and model’s estimate is 92% and the residuals fall along the 45-degree line of the normal quantile plot. This shows that our RF model fits the data well in addition to offering strong out-of-sample prediction as shown in Table 3.

**Fig 9:**
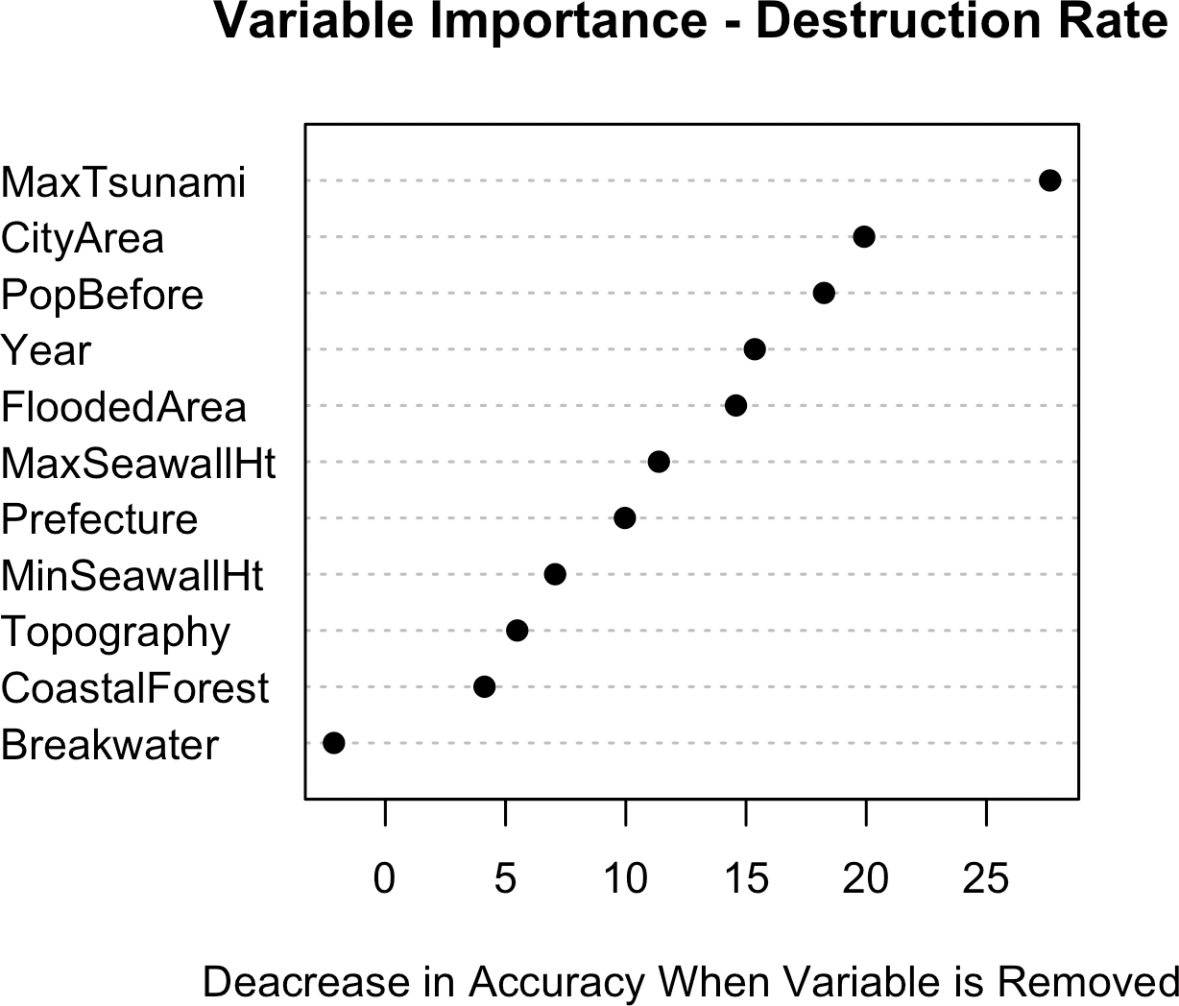
Importance of each of the explanatory variables used in the RF model for predicting Destruction Rates.

Fig 9 shows the relative importance of each of the explanatory variables used in the RF model of destruction rate. The importance of population is self-explanatory, being an indicator of the presence of vulnerable housing. The influence of tsunami height and flooded area on destruction rate is also obvious. City area is important because it is necessary for describing what fraction of each municipality lies inside, and what fraction outside, the inundation zone. Year represents many factors related to destruction rate, such as building codes and land use planning. Protective measures (seawall height and coastal forest area) show less importance in the model than the tsunami itself, but are nonetheless significant. The presence of a bay mouth breakwater is insignificant in the model because of the lack of data points (a bay mouth breakwater was only present in 3 of the municipalities investigated in 2011, with none present during earlier events). The variables of prefecture and topography are closely related to one another, and neither has a strong effect on the model result.

#### Partial dependence plots

**Fig 10:**
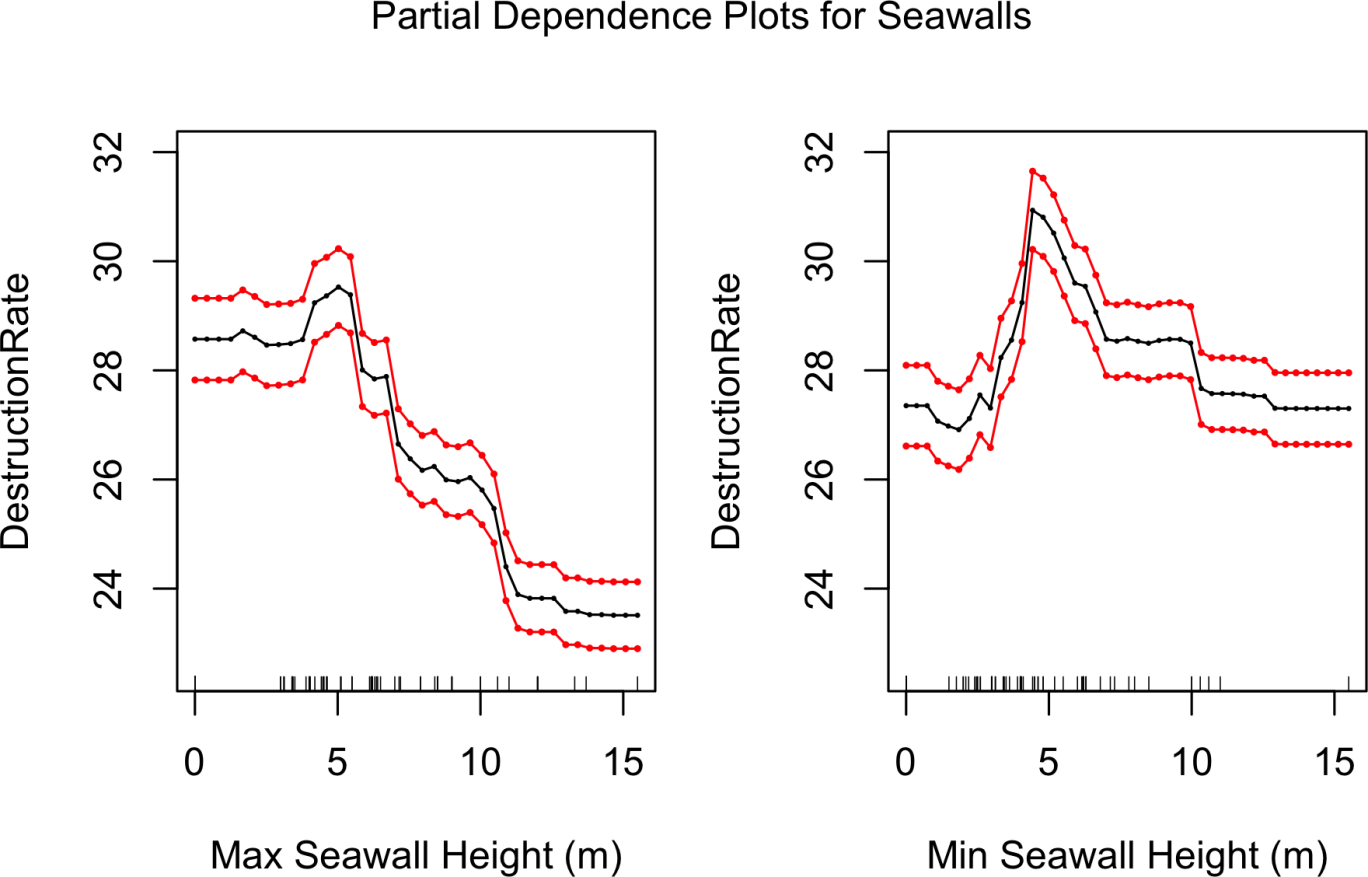
Partial dependence plots of minimum and maximum seawall heights and mean destruction rate. The red lines represent bootstrapped confidence intervals around model estimates.

**Fig 11:**
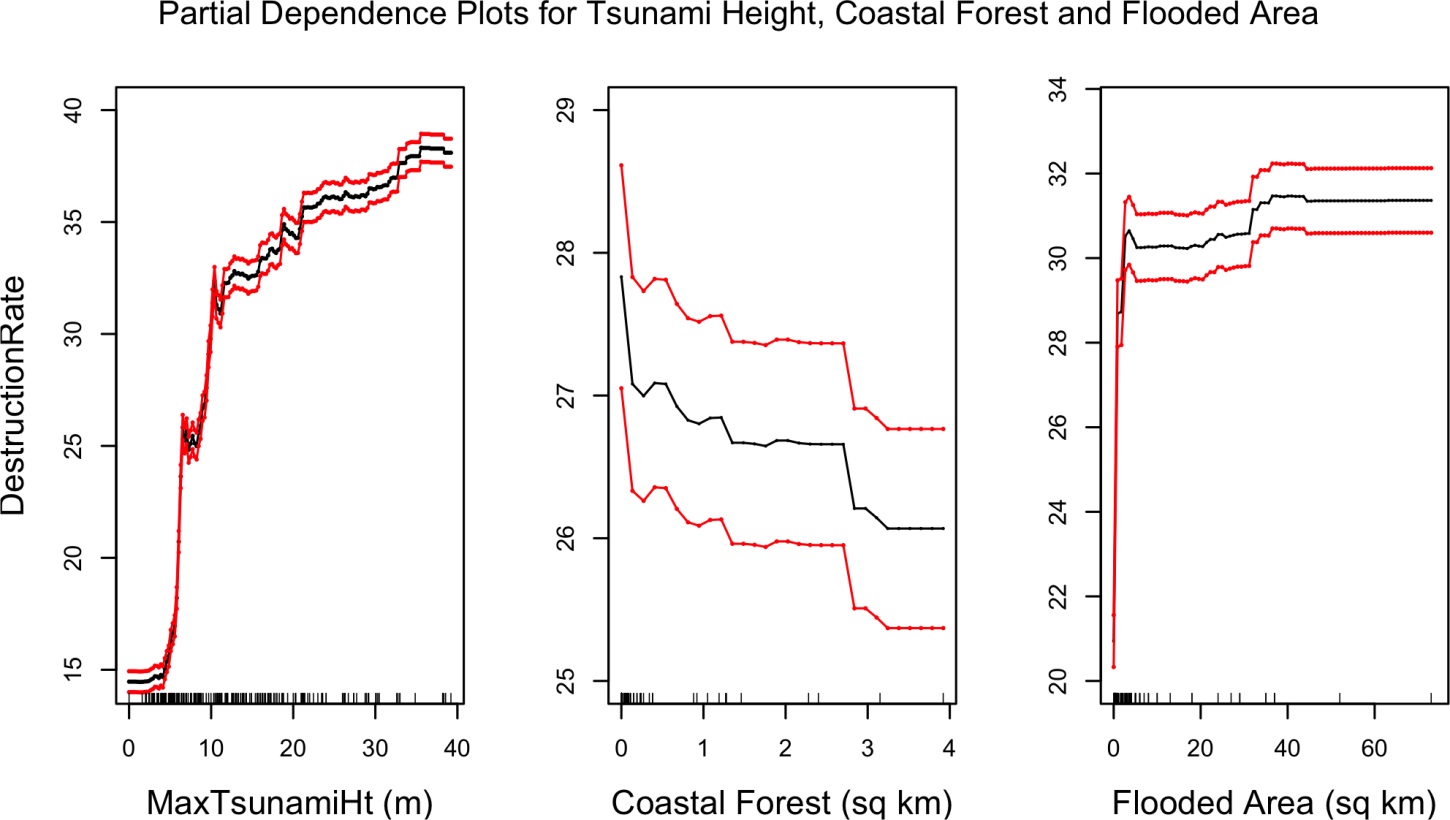
Partial dependence plots of maximum tsunami height, coastal forest area, and flooded areas with mean destruction rate. The red lines represent bootstrapped confidence intervals around model estimates.

Since our best model (RF) is non-parametric, to examine the relationship between each covariate and response we use partial dependency plots as discussed in the Methods section. Fig 10 shows the partial dependencies between seawall heights and mean destruction rate. It is interesting to see that the destruction rates peak at seawall heights of 5 meters, afterwhich there is a decreasing trend suggesting that seawalls higher than 5 meters have been effective in reducing building damage. This is a similar conclusion to that reached with Fig 7. Fig 11 shows the partial dependencies to maximum tsunami height, coastal forests area, and flooded area. As expected larger tsunami heights are associated with higher destruction rates. Coastal forest area shows an inverse relationship with destruction rate, indicating that forests either mitigate tsunami damage, or prevent development where they are planted which in turn reduces what is there to be damaged. It must be kept in mind that coastal forests did not always mitigate death and destruction rates. A case in point is Rikuzentakata, which had a large coastal forest, but in 2011 saw this forest destroyed, together with a large portion of the city’s buildings [38]. However, over all 4 historical events and throughout all municipalities investigated, larger forest areas correlate with lower damage rates.

### Death Rates

This section summarizes the predictive performance of a series of models fitted to our data. In this case the response is death rates and the explanatory variables include the year of the event, number of dwellings before the event, maximum tsunami height, municipal area, coastal forest area, presence of a bay-mouth breakwater, maximum and minimum seawall height, flooded area, prefecture and topography. In order to deal with the missing data in our input variables, we used the Multivariate Imputation by Chained Equations (MICE) algorithm.

#### Out-of-sample prediction errors

The methods of Bayesian Additive Regression Trees (BART) and Random Forest (RF) out-performed all other models in terms of their predictive accuracy. Even though the errors look slightly less for BART, the difference between BART and RF is not statistically significant. It can be seen from Table 4 that the errors associated with BART and RF are substantially lower than the ‘mean-only (aka Null)’ model suggesting the effectiveness of the models in capturing the variability in death rates. Even though BART’s out-of-samples errors were slightly lower than RF, the RF model fitted our data better (with correlation of model’s estimate and the observed values being 94% for RF as opposed to 90% in BART). Given that differences between the predictive accuracies of RF and BART were not statistically significant and better fit of the RF model, we selected RF as our final best model.

**Table 4:** Out-of-sample predictive accuracy across different models.

Model	MSE	SE	MAE	SE
Null	204.0	28.3	8.8	0.4
BART	**107.8**	**16.6**	**5.1**	**0.3**
RF	**121.0**	**19.8**	**5.5**	**0.3**
MARS1	149.8	17.8	7.4	0.3
MARS2	156.4	19.8	7.4	0.3
MARS3	139.2	23.8	6.8	0.4
MARS5	152.6	20.3	6.8	0.4
SVM	159.7	25.8	5.8	0.4
GBM	189.9	27.2	8.4	0.4

**Fig 12:**
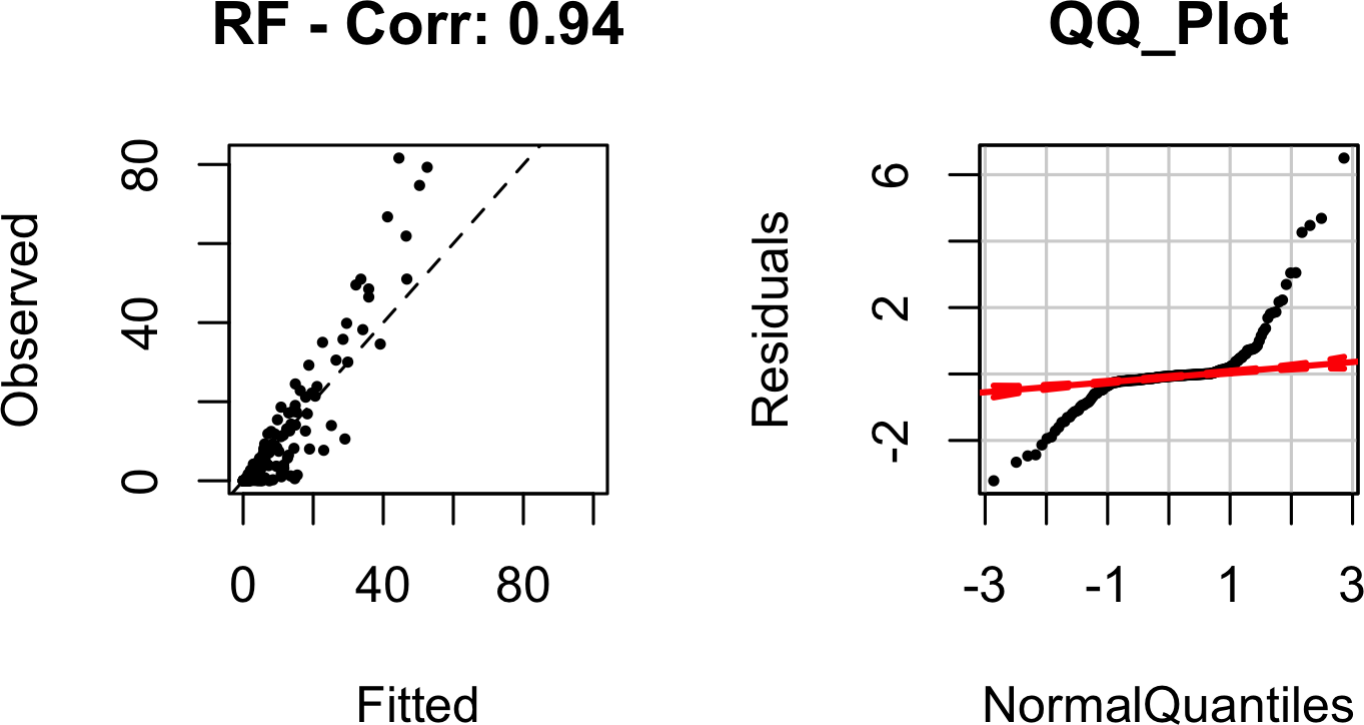
Plot of observed versus fitted values of death rate for methods of RF. The red dashed lines in the QQ-plot represent 95% confidence intervals.

Fig 12 summarizes our model’s fit. It can be seen that our model tends to under-estimate death-rates for values above 30% and lean towards over-estimation for values below 20%. The normal quantile plot also shows non-ideal tail behavior, suggesting that there are possibly other key variables (e.g. effectiveness of warning systems) that are essential for understanding death rates and are missing from our models.

**Fig 13:**
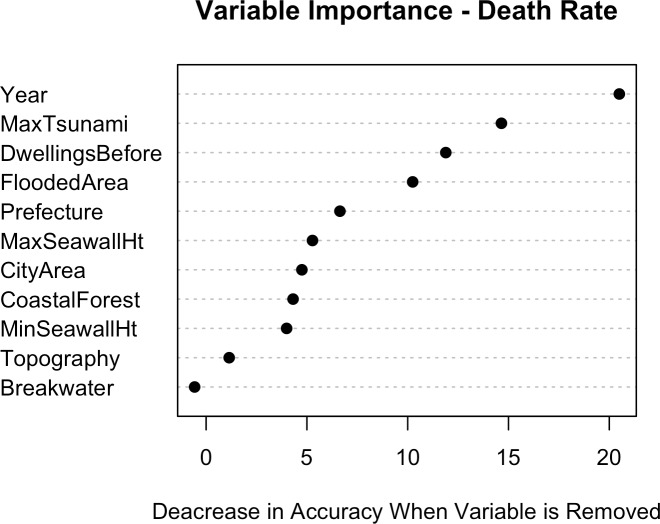
Importance of each of the explanatory variables used in the RF model for predicting Death Rates.

Fig 13 shows that, as with the case of destruction rate, year is a very important explanatory variable. In this case, Year is a proxy for factors such as the presence of tsunami warning systems, evacuation centers, evacuation training and drills, access to warning media, and the different physical characteristics of each earthquake, including time of day, magnitude of ground shaking before each tsunami, and location (near-field vs. far-field) of the tsunami source. Seawall height is less important to the prediction of death rate than to destruction rate, indicating that people tend to evacuate regardless of the presence of these protective measures (people can evacuate, but buildings can’t). However, this trend may also be due to the fact that the model predicts destruction rate better than it does death rate.

#### Partial dependence plots

**Fig 14:**
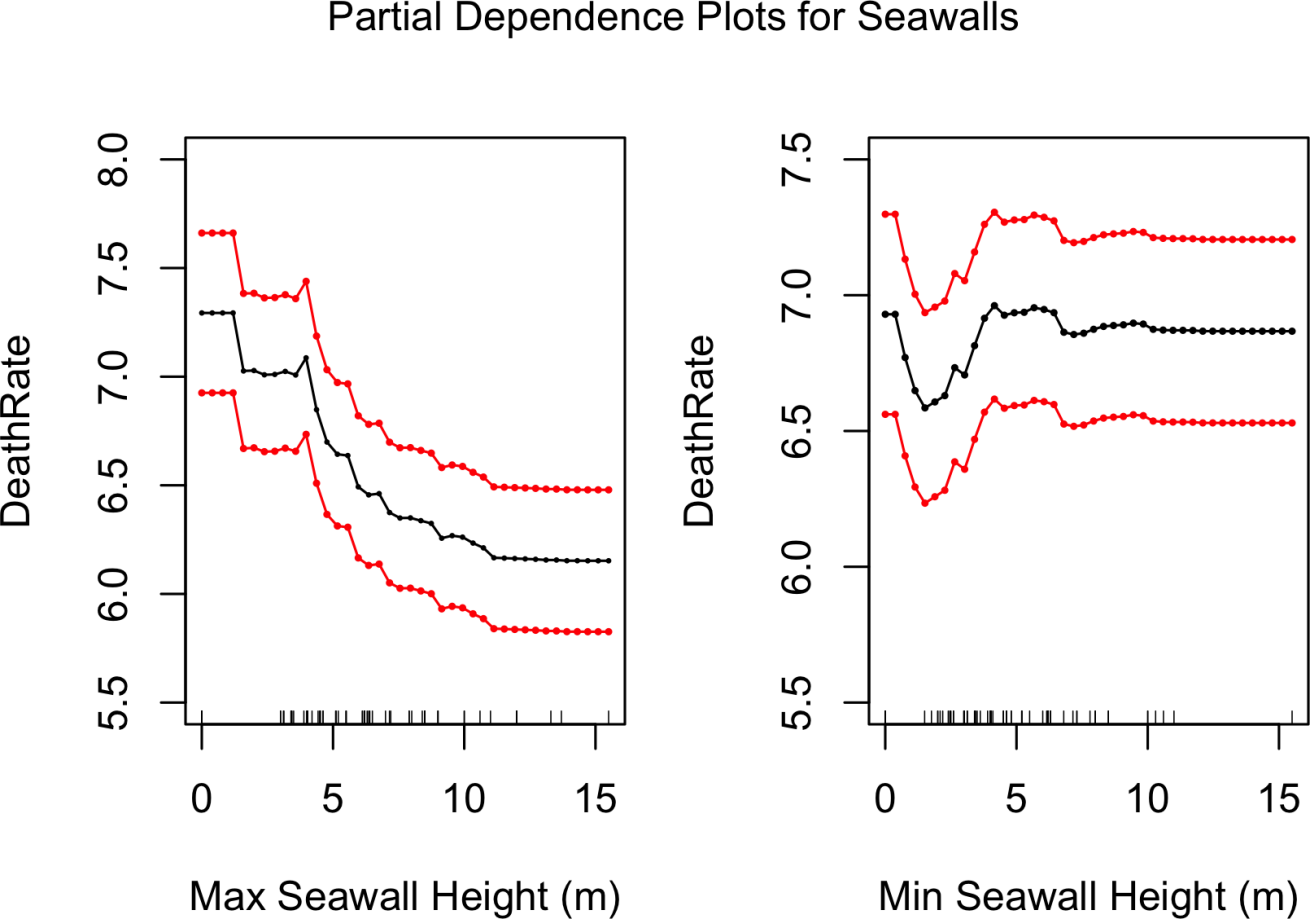
Partial dependencies of minimum and maximum seawall heights with death rates. The red lines represent bootstrapped confidence intervals around model estimates.

**Fig 15:**
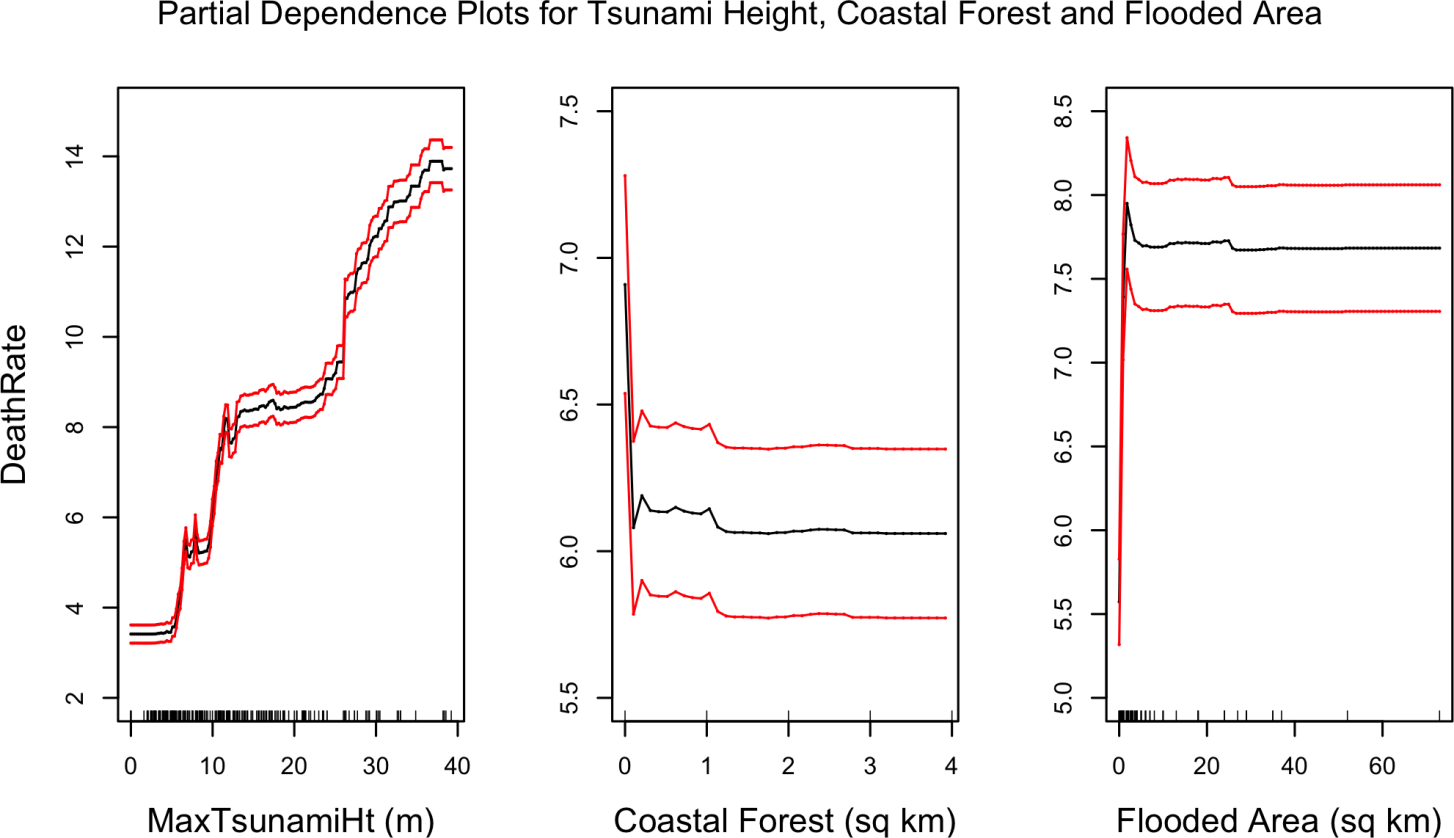
Partial dependencies between maximum tsunami height, coastal forest area, and flooded areas with mean death rates. The red lines represent bootstrapped confidence intervals around model estimates.

Fig 14 shows the partial dependencies of seawall heights with death rates. Just as in destruction rates (Fig 10), it can be seen than large seawalls are associated with lower death rates. Fig 10 showed that relatively small seawalls (5 m high) exacerbated destruction; a similar trend appears in Fig 14 with death rates as well, but this trend is not as pronounced as it is with destruction rates. Also in line with destruction rates, Fig 15 shows that higher death rates are associated with larger tsunami heights and flooded areas, and that larger coastal forests are associated with lower death rates.

## Discussion and Recommendations

The significant outcome of this work is seen in the partial dependence of mortality and destruction rate on seawall height and coastal forest area (Figs 7, 10, 11, 14 and 15). Large seawalls are shown to have been effective at reducing both mortality and damage rate, but smaller seawalls (around 5 m high) showed no effectiveness in reducing impact. Coastal forests are shown to have been effective at reducing both mortality and damage rates, though this study could not determine if this was due to the physical mitigating effect of forests, or due to the prevention of development in tsunami-prone areas after these forests were planted. Furthermore, seawall height and coastal forest area had a stronger effect on predicting destruction rates than on death rates, hinting that people’s decisions to evacuate were not dependent on the presence of protective measures, though this inference needs to be tempered by the fact that the model predicted destruction rate much better than death rate overall.

In order to concentrate on the effectiveness of man-made coastal defense structures in mitigating death and damage, the present work neglects the importance of other factors. [39] points out that mortality is determined by other factors as well: water rise rate, proximity to dike breach, flow speed, and warning time. [14] shows the importance of community cohesiveness in reducing mortality, while [40] stresses the importance of education, warning, and evacuation over physical flood mitigation measures. Nonetheless, the historical data used in the present work clearly show the effect of seawalls themselves, and can be used to end the debate on whether these walls reduced or exacerbated mortality and damage.

## Supporting Information

S1 Table Historical tsunami and damage data. (XLSX)

## References

[1] Muhari A, Imamura F, Suppasri A, Mas E. Tsunami arrival time characteristics of the 2011 East Japan Tsunami obtained from eyewitness accounts, evidence and numerical simulation. Journal of Natural Disaster Science, 34(1), 91–10. 2012.

[2] Shuto N, Fujima K. A Short History of Tsunami Research and Countermeasures in Japan. Proceedings of the Japan Academy, Series B, Physical and Biological Sciences. 2009; 85(8), 267–275. 10.2183/pjab.85.267 PMC3621565 19838008

[3] Mori N, Takahashi T, Yasuda T, Yanagisawa H. Survey of 2011 Earthquake Tsunami Inundation and Run-up. Geophysical Research Letters. 2011; 38(7), 10.1029/2011GL049210

[4] Ando A, Sasaki Y, Akatani R, Miura T. A Historical Study on Tsunami Disaster Prevention of the Coastal Area in Iwate. Journal of the Japan Society of Civil Engineers. 2000; 639, IV–46, 1–11. Japanese.

[5] JMA. Improvement of the Tsunami Warning System. Japan Meteorological Agency. 2012. http://www.jma.go.jp/jma/kishou/minkan/koushu120518/shiryou4.pdf. Japanese.

[6] Harada K, Imamura F. Effects of Coastal Forest on Tsunami Hazard Mitigation–a Preliminary Investigation. Tsunamis: Case Studies and Recent Developments, K. Satake (ed.), Advances in Natural and Technological Hazards Research. 2005; Vol. 23. Springer. Pp279-292.

[7] Iwate Prefecture. Great East Japan Earthquake and Tsunami Reconstruction Plan. Basic Reconstruction Plan. 2011. http://www2.pref.iwate.jp/~hp0212/fukkou_net/pdf_doc/kihonkeikaku_english.pdf

[8] Miyagi Prefecture. Earthquake Disaster Recovery Plan. 2011. http://www.pref.miyagi.jp/uploaded/attachment/36634.pdf

[9] Koshimura S, Shuto N. Response to the 2011 Great East Japan Earthquake and Tsunami disaster. Phil. Trans. R. Soc. A. 2015; 373.2053: 20140373 10.1098/rsta.2014.0373 26392623

[10] Japan Times. Japan drafts new five-year blueprint for tsunami reconstruction. 2016. http://www.japantimes.co.jp/news/2016/01/19/national/japan-drafts-new-five-year-blueprint-tsunami-reconstruction/

[11] New York Times. Seawalls Offered Little Protection against Tsunami’s Crushing Waves. March 13, 2011 Asia-Pacific. 2011. http://www.nytimes.com/2011/03/14/world/asia/14seawalls.html?pagewanted=all&_r=0

[12] The Economist. The Great Wall of Japan. 2014. http://www.economist.com/news/asia/21604200-tsunami-protectionor-boondoggle-builders-great-wall-japan

[13] Kahoku Shimpo. Bearing the costs of reconstruction: Reigniting the debate over seawalls. 2015. http://www.kahoku.co.jp/tohokunews/201506/20150601_13014.html (in Japanese).

[14] Aldrich DP, Sawada Y. The Physical and Social Determinants of Mortality in the 3.11 Tsunami. Social Science & Medicine. 2015; 124: 66–75. 25461863 10.1016/j.socscimed.2014.11.025

[15] Tomita T, Yeom GS, Ayugai M, Niwa T. Breakwater Effects on Tsunami Inundation Reduction in the 2011 off the Pacific Coast of Tohoku Earthquake. Journal of the Japan Society of Civil Engineers, Ser. B2 (Coastal Engineering). 2012; 68(2), I_156-I_160. Japanese.

[16] Huffington Post. How Fudai, Japan Defied the Tsunami Devastation. 2011. http://www.huffingtonpost.com/2011/05/13/fudai-japan-tsunami-_n_861534.html?

[17] Cyranoski D. Rebuilding Japan: After the deluge. Nature. 2012; 483, 141–143 10.1038/483141a 22398535

[18] MLITT. Political map boundary data. Ministry of Land Infrastructure Transport and Tourism. 2015. http://nlftp.mlit.go.jp/ksj/. Japanese.

[19] MLITT. Damage to Seawalls due to Earthquake and Tsunami, and Important Points for Design of Tsunami-Resistant Structures. Ministry of Land, Infrastructure, Transport, and Tourism. 2013a. http://www.mlit.go.jp/common/001020132.pdf. Japanese.

[20] PWRI. Quick Report on Damage to Infrastructres by the 2011 off the Pacific Coast of Tohoku Earthquake. Technical Note of the National Institute for Land and Infrastructure Management, No. 646. Technical Note of Public Works Research Institute, No. 4202. 2011.

[21] Iwate Prefecture. Reconstruction and Maintenance of Tsunami Disaster Prevention Facilities. 2015b. http://www.pref.iwate.jp/kasensabou/kasen/fukkyuu/tsunami/index.html (in Japanese).

[22] Mori N, Cox DT, Yasuda T, Mase H. Overview of the 2011 Tohoku Earthquake Tsunami Damage and its Relation to Coastal Protection along the Sanriku Coast. Earthquake Spectra. 2013; 29(S1): S127–S143.

[23] Yun NY, Hamada M. Evacuation behavior and fatality rate of residents during the 2011 Great East Japan earthquake and tsunami. Earthquake Spectra. 2014; 10, 1193.

[24] Takahashi M, Matsushita N. What differentiated local casualties caused by the tsunami? Journal of Geography (Chigaku Zasshi). 2015; 124(2) 193–209. Japanese.

[25] Bricker JD, Nakayama A. Contribution of trapped air, deck superelevation, and nearby structures to bridge deck failure during a tsunami. Journal of Hydraulic Engineering, ASCE. 2014; 40 (5), 05014002-1-7.

[26] Shimozono T, Cui H, Pietrzak JD, Fritz HM, Okayasu A, Hooper AJ. Short Wave Amplification and Extreme Runup by the 2011 Tohoku Tsunami. Pure Appl. Geophys., 171(12):3217–3228, 10.1007/s00024-014-0803-1 2014.

[27] Fritz HM, Kongko W, Moore A, McAdoo B, Goff J, Harbitz C, et al Extreme runup from the 17 July 2006 Java tsunami, Geophys. Res. Lett., 34, L12602, doi: 10.1029/2007GL029404. 2007

[28] Hill EM, Borrero JC, Huang Z, Qiu Q, Banerjee P, Natawidjaja DH, et al The 2010 Mw 7.8 Mentawai earthquake: Very shallow source of a rare tsunami earthquake determined from tsunami field survey and near-field GPS, J. Geophys. Res. Solid Earth, 117, B06402, doi: 10.1029/2012JB009159. 2012

[29] Nelder JA, Wedderburn RWM Generalized Linear Models. Journal of the Royal Statistical Society. Series A (General), 1972; pp.370–384.

[30] Hastie T, Tibshirani R. Generalized additive models: some applications. Journal of the American Statistical Association, 1987; 82(398): 371–386.

[31] Friedman JH. Multivariate adaptive regression splines. The annals of statistics. 1991: pp.1–67.

[32] Chipmman HA, George EI, McColluch RE. BART: Bayesian Additive Regression Trees, Ann. Appl. Stat. 2010; 4(1):266–298.

[33] Breiman L. Random forests. Machine learning. 2001 10 1;45(1):5–32.

[34] Smola A., Vapnik V. Support vector regression machines. Advances in neural information processing systems. 1997; 9:155–161.

[35] Vapnik V, Golowich SE, Smola A. Support vector method for function approximation, regression estimation, and signal processing. In Advances in Neural Information Processing Systems, 1996; 9.

[36] Friedman JH. Greedy function approximation: a gradient boosting machine. Annals of statistics. 2001:1189–1232.

[37] Hasti T, Tibshirani R, Friedman J. The Elements of Statistical Learning; Data Mining, Inference and Prediction, 1st ed., New York: Springer, pp. 283–287 & 267–270, 2001.

[38] Liu H, Shimozono T, Takagawa T, Okayasu A, Fritz HM, Sato S, Tajima Y. The 11 March 2011 Tohoku Tsunami Survey in Rikuzentakata and Comparison with historical Events. Pure Appl. Geophys., 170(6–8):1033–1046, doi: 10.1007/s00024-012-0496-2. 2013

[39] Jonkman SN, Vrijling AJK, Vrouwenvelder ACWM. Methods for the estimation of loss of life due to floods: a literature review and a proposal for a new method. Nat Hazards. 2008; 46:353–389

[40] Fritz HM, Blount CD, Thwin S, Thu MK, Chan N. Cyclone Nargis storm surge in Myanmar, Nature Geoscience 2(7):448–449, doi: 10.1038/ngeo558. 2009

[41] E-Stat. Dwellings by Occupancy Status. Portal Site of Official Statistics of Japan. 2009. http://www.e-stat.go.jp/SG1/estat/List.do?bid=000001023943&cycode=0. Japanese.

[42] FDMA. Extent of Inundation and Flooded Area of each City, Town, and Village. Fire and Disaster Management Agency. 2013. http://www.fdma.go.jp/neuter/about/shingi_kento/jishin_tsunami/02/sanko-3.pdf. Japanese.

[43] GSI. Countrywide Table of Municipal Land Areas. Geospatial Information Authority of Japan. 2012. http://www.gsi.go.jp/KOKUJYOHO/MENCHO-title.htm. Japanese.

[44] Hayashi N. Damage to Taro due to the Earthquake Tsunami. Graduation thesis from the Tokyo University of Science, Tsujimoto Laboratory. 2012. http://www.rs.kagu.tus.ac.jp/tujimoto/2012hayashi.pdf. Japanese.

[45] Interior Ministry. Report of Damage to the Towns and Villages Affected by the Sanriku Tsunami. Ministry of the Interior, City Planning Division. The Tokyo Institute for Municipal Research Digital Archives. 1934. Japanese.

[46] Ishinomaki City. Casualties, Population, Damage. 2015. https://www.city.ishinomaki.lg.jp/cont/10106000/7253/20141016145443.html, http://www.city.ishinomaki.lg.jp/cont/10102000/0040/3914/jinkoumenseki1506.pdf, https://www.city.ishinomaki.lg.jp/cont/10181000/7742/01_dai1syou.pdf. Japanese.

[47] Iwate Prefecture. Survey of the Condition of Iwate Prefecture after the Kaisho. Library of Nobuo Shuto. 1896. Japanese.

[48] Iwate Prefecture. Report of Damage to Civil Works due to the Earthquake Tsunami. Iwate Prefecture, Dept. of Civil Works. Library of Nobuo Shuto. 1936. Japanese.

[49] Iwate Prefecture. Report of Reconstruction after the Chile Earthquake Tsunami. Iwate Prefecture, Dept. of Economics, Dept. of Agriculture and Forestry, and Dept. of Civil Works. Library of Nobuo Shuto. 1969. Japanese.

[50] Iwate Prefecture. Iwate Prefecture Expected Tsunami Inundation Maps. 2004. Japanese.

[51] Iwate Prefecture. Record of the Great East Japan Earthquake and Tsunami in Iwate Prefecture. 2013. http://www2.pref.iwate.jp/~bousai/kirokushi/2013kirokushi.html. Japanese.

[52] Iwate Prefecture. Iwate Prefecture Population Movement Annual Report from 1951 Onwards. 2015. http://www3.pref.iwate.jp/webdb/view/outside/s14Tokei/keywordKekka.html;jsessionid=032CDEB9540AE068F7A763DE3B377FA5?keyword=%25E4%25B8%2596%25E5%25B8%25AF&tyosaCategory=%25E3%2581%259B. Japanese.

[53] JSCE. Summary of Damage in Areas Surveyed. Japan Society of Civil Engineers. 2011. http://committees.jsce.or.jp/2011quake/system/files/2011_2nd_0017.pdf. Japanese.

[54] Kamaishi City. Status of Damage due to the Great East japan Earthquake and Tsunami. 2011. http://www.city.kamaishi.iwate.jp/. Japanese.

[55] Kesennuma City. Damage Status. 2011. http://www.city.kesennuma.lg.jp/www/contents/1308917412557/files/hukko1shiryo5.pdf. Japanese.

[56] Kesennuma Survey Group. 1960 Chile Earthquake and Sanriku Tsunami Memorial Report. Library of Nobuo Shuto. 1961. Japanese.

[57] MAFF. Data for the Town of Iwaizumi. Ministry of Agriculture, Forestry, and Fisheries. 2013a. http://www.machimura.maff.go.jp/machi/contents/03/483/index.html. Japanese.

[58] MAFF. Data for the Town of Tanohata. Ministry of Agriculture, Forestry, and Fisheries. 2013b. http://www.machimura.maff.go.jp/machi/contents/03/484/index.html. Japanese.

[59] MAFF. Data for the Town of Fudai. Ministry of Agriculture, Forestry, and Fisheries. 2013c. http://www.machimura.maff.go.jp/machi/contents/03/485/index.html. Japanese.

[60] MAFF. Data for the Town of Onagawa. Ministry of Agriculture, Forestry, and Fisheries. 2013d. http://www.machimura.maff.go.jp/machi/contents/03/581/index.html. Japanese.

[61] Minamisanriku Town. Population and number of buildings, Reconstruction Plan. 2011. http://www.town.minamisanriku.miyagi.jp/index.cfm/10,801,56,239,html#togura, http://www.pref.miyagi.jp/uploaded/attachment/88091.pdf. Japanese.

[62] Miyagi Prefecture. Survey of Miyagi Prefecture after the Kaisho. Library of Nobuo Shuto. 1903. Japanese.

[63] Miyagi Prefecture. Chile Earthquake Tsunami Damage Aid Report. Library of Nobuo Shuto. 1961. Japanese.

[64] Miyagi Prefecture. Status of Damage due to the Great East Japan Earthquake and Tsunami. 2014. http://www.pref.miyagi.jp/site/ej-earthquake/km-higaizyoukyou.html. Japanese.

[65] Miyako City. Damage Summary, Plan for the Reconstruction of Taro. 3^rd^ Meeting of the Taro Reconstruction Planning Commission. 2011. http://www.city.miyako.iwate.jp/data/open/cnt/3/4864/1/03_31-70.pdf, http://www.city.miyako.iwate.jp/data/open/cnt/3/713/1/03-06siryo4_image.pdf. Japanese.

[66] MLITT. Specification of the Heights of Coastal Levees Along the Miyagi Coast. 2013. http://www.thr.mlit.go.jp/Bumon/B00097/K00360/taiheiyouokijishinn/kaigann/kaigann2.pdf. Japanese.

[67] Natori City. Damage Record. 2014. http://www.city.natori.miyagi.jp/content/download/29726/173138/file/natorishi-kiroku-all.pdf. Japanese.

[68] Onagawa Town. Damage Map. 2015. http://www.town.onagawa.miyagi.jp/hukkou/pdf/iinkai/01_meeting/01_meeting_appendix3-2.pdf. Japanese.

[69] Ofunato City. Personal communication. 2015.

[70] Reconstruction Agency. Reconstruction Plan (for each municipality). 2011. http://www.reconstruction.go.jp/topics/cityname_setto.pdf. Japanese.

[71] Reconstruction Agency. Plan for the Reconstruction of Coastal Protection Facilities in Kuji City, Iwate Prefecture. 2013a. http://www.reconstruction.go.jp/topics/kuzishi_setto.pdf. Japanese.

[72] Reconstruction Agency. Plan for the Reconstruction of Coastal Protection Facilities in Otsuchi City, Iwate Prefecture. 2013b. http://www.reconstruction.go.jp/topics/212iwate09otsuchi0611.pdf. Japanese.

[73] Rikuzentakada City. Damage Status. 2015. http://www.city.rikuzentakata.iwate.jp/shinsai/oshirase/hazard1.pdf. Japanese.

[74] Sendai City. Damage Report. 2011. http://www.city.sendai.jp/shinsai/kirokushi/05_Chapter2-s.pdf. Japanese.

[75] Statistics Japan. Historical Japanese Census Data for 1935, 1960, and 2010. 2015. http://www.stat.go.jp/data/kokusei/2010/. Japanese.

[76] Tani K. Casualty rates per neighborhood. Saitama University Dept. of Geography research report. 2012. http://sucra.saitama-u.ac.jp/modules/xoonips/download.php/KY-AN00162484-32-01.pdf?file_id=32990. Japanese.

[77] Tani K. Past and present map comparison website. Saitama University. 2015. http://ktgis.net/kjmapw/. Japanese.

[78] Tohoku University. Japan Tsunami Trace Database. 2015. http://153.142.0.53/tsunami/mainframe.php. Japanese.

[79] Watanabe H. Comprehensive List of Tsunamis to Hit the Japanese Islands. Second Edition. University of Tokyo Press. ISBN978-4-13-061113-8. 1998. Japanese.

[80] Watari City. Personal Communication. 2015.

[81] Wikipedia. Entries for each historic municipality in Iwate and Miyagi Prefectures. 2015. http://ja.wikipedia.org/wiki/[town name in kanji]. Japanese.

